# The Link Between Tau and Insulin Signaling: Implications for Alzheimer’s Disease and Other Tauopathies

**DOI:** 10.3389/fncel.2019.00017

**Published:** 2019-02-05

**Authors:** Rafaella Araujo Gonçalves, Nadeeja Wijesekara, Paul E. Fraser, Fernanda G. De Felice

**Affiliations:** ^1^Centre for Neuroscience Studies, Queen’s University, Kingston, ON, Canada; ^2^Tanz Centre for Research in Neurodegenerative Diseases, University of Toronto, Toronto, ON, Canada; ^3^Department of Medical Biophysics, University of Toronto, Toronto, ON, Canada; ^4^Department of Psychiatry, Queen’s University, Kingston, ON, Canada; ^5^Institute of Medical Biochemistry Leopoldo de Meis, Federal University of Rio de Janeiro, Rio de Janeiro, Brazil

**Keywords:** insulin resistance, tau protein, MAPT, Alzheimer’s disease, tauopathy, cognitive decline, diabetes

## Abstract

The microtubule-associated protein tau (MAPT) is mainly identified as a tubulin binding protein essential for microtubule dynamics and assembly and for neurite outgrowth. However, several other possible functions for Tau remains to be investigated. Insulin signaling is important for synaptic plasticity and memory formation and therefore is essential for proper brain function. Tau has recently been characterized as an important regulator of insulin signaling, with evidence linking Tau to brain and peripheral insulin resistance and beta cell dysfunction. In line with this notion, the hypothesis of Tau pathology as a key trigger of impaired insulin sensitivity and secretion has emerged. Conversely, insulin resistance can also favor Tau dysfunction, resulting in a vicious cycle of these events. In this review article, we discuss recent evidence linking Tau pathology, insulin resistance and insulin deficiency. We further highlight the deleterious consequences of Tau pathology-induced insulin resistance to the brain and/or peripheral tissues, suggesting that these are key events mediating cognitive decline in Alzheimer’s disease (AD) and other tauopathies.

## Introduction

Tau protein was first isolated from the porcine brain as a factor essential for microtubule assembly (Weingarten et al., 1975). Eleven years after its first characterization, Tau was shown to be a component of paired helical filaments (PHFs), the main constituents of Neurofibrillary tangles (NFTs) in the Alzheimer’s disease (AD) brain (Grundke-Iqbal et al., 1986). The need to understand the role of Tau in AD and other tauopathies resulted in years of studies focusing on the pathological role of this protein and therefore, currently there exists a gap in what we know about the physiological functions of Tau. Bridging this gap will be instrumental to better comprehend the contribution of this protein to disease states.

Tau is a natively soluble and unfolded protein that undergoes a variety of post-translational modifications (PTMs), which directly or indirectly modulates its physiological and pathological functions. Phosphorylation is the most common PTM described for Tau, and Tau has 85 different phospho-sites (Guo et al., 2017). A proper balance in the number of phosphorylated and dephosphorylated residues of Tau favors its classical physiological role in neurite outgrowth, axonal transport and microtubule dynamics. In addition, due to its scaffolding property, Tau can bind to a variety of proteins, therefore impacting multiple physiological functions (Meier et al., 2015; Suzuki and Kimura, 2017; Zhou et al., 2017; Stefanoska et al., 2018). Many of these interactions still remain to be uncovered. Under pathological conditions, an imbalance between the activities of kinases and phosphatases originates high levels of abnormally phosphorylated Tau, which have reduced affinity to microtubules and results in cytoskeleton destabilization. In addition, Tau hyperphosphorylation induces its missorting from axons into somatodendritic compartments, which is linked to synaptic dysfunction and cell death (Guo et al., 2017). Highly phosphorylated Tau self-assembles into PHFs and then NFTs, insoluble dense intracellular lesions considered a key hallmark of important human neurodegenerative disorders, such as AD, frontotemporal dementia (FTD), and other Tauopathies (Goedert and Spillantini, 2011).

In this review article, we discuss recent findings suggesting a novel physiological function for Tau protein as a regulator of insulin signaling in the brain and in peripheral tissues, and we further discuss the possible implications of Tau pathology-induced insulin resistance to cognition. A thorough understanding of the mechanisms linking Tau and insulin signaling becomes important when elucidating the increased risk of dementia associated with diabetes.

## Tau Protein and Insulin Signaling in the Brain

Insulin, the main regulator of glucose homeostasis and metabolism, is also crucial for a variety of different brain functions such as synaptic plasticity, learning and memory (Duarte et al., 2012; Fernandez and Torres-Alemán, 2012; De Felice, 2013; Kleinridders et al., 2014). In addition to its classical role as a microtubule-stabilizer, Tau acts as a scaffolding protein and interacts with components of the insulin signaling pathway in the brain. The N-terminal portion of Tau can bind to Src homology 3 (SH3) domains of Src family tyrosine kinases, which include domains from the p85alpha subunit of phosphatidylinositol 3-kinase (PI3K), a key protein in the insulin signaling pathway (Reynolds et al., 2008). Co-immunoprecipitation studies using wild-type (WT) mouse brain tissue and N1E115 cells transfected with WT human Tau reported that Tau binds to phosphatase and tensin homolog protein (PTEN), a negative regulator of insulin transduction that catalyzes the dephosphorylation of phosphatidylinositol (3,4,5)-triphosphate (PIP3) resulting in the formation of phosphatidylinositol (4,5)-diphosphate (PIP2). Therefore, by interacting with PTEN, Tau reduces its activity and favors insulin signaling (Marciniak et al., 2017). These studies raise the possibility that the ability of insulin to maintain proper brain activity depends on Tau and, conversely, pathological forms of Tau could be deleterious due to a loss of proper protein function. This idea was corroborated by a study showing that Tau deletion impaired hippocampal insulin sensitivity (Marciniak et al., 2017). Tau ablation in mice further resulted in deficits in long-term potentiation and contextual fear conditioning responses (Ahmed et al., 2014). In another study, the knockout (KO) of Tau in a mouse of B6129PF1/J background strain induced age-dependent defects in short-term memory and in synaptic plasticity (Biundo et al., 2018).

Impaired brain insulin signaling has been consistently associated with cognitive decline in animal models and in humans (Moloney et al., 2010; Bomfim et al., 2012; Craft et al., 2012, 2017; Lourenco et al., 2015; Benedict and Grillo, 2018). Under pathological conditions, loss of physiological function when Tau is hyperphosphorylated may trigger impairments in insulin signaling that will negatively impact the brain. The ability of Tau to interact with SH3 domains inversely correlates with its phosphorylated levels (Reynolds et al., 2008), suggesting that the scaffolding properties of Tau are regulated by its phosphorylation status. Moreover, increased Tau phosphorylation and decreased insulin signaling was observed in the hippocampus of middle-aged (17 months) rats, and it was linked to deficits in spatial learning (Kuga et al., 2018). In another study, hyperphosphorylated Tau-containing neurons from insoluble fractions of post-mortem AD and other tauopathies were shown to contain insulin accumulated as oligomers (Rodriguez-Rodriguez et al., 2017). In this study, the authors showed that the intraneuronal accumulation of insulin was associated with impairments in insulin signaling, characterized by reduced neuronal insulin receptor expression, as well as decreased downstream components of the insulin signaling pathway, such as phosphorylated protein kinase B (AkT). Studies using animal models that develop Tau pathology are warranted to determine whether impaired synaptic function and insulin signaling are a result of Tau loss of function due to Tau aggregation.

The loss of function hypothesis for Tau toxicity also extends to the findings in clinics. Reduced brain levels of soluble Tau are characteristic of the most common pathological variant of FTD, known as dementia lacking distinctive histopathology (DLDH). In these cases, reduced soluble Tau and the absence of insoluble or fibrillary Tau inclusions are associated with disease severity (Zhukareva et al., 2003). Proteomic analysis has found a high correlation between reduced Tau protein levels, synaptic impairment and reactive gliosis in FTD cases (Papegaey et al., 2016).

Although the influence of Tau pathology on insulin signaling is not completely understood, it is known that insulin resistance can induce Tau hyperphosphorylation and cognitive decline in human and in animal models (Deng et al., 2009; El Khoury et al., 2014; Yarchoan et al., 2014; Benedict and Grillo, 2018). Systemic insulin resistance was linked to poorer performance on cognitive tests and higher levels of cerebrospinal fluid (CSF) phosphorylated and total Tau in cognitively normal individuals (Laws et al., 2017). Conversely, systemic insulin resistance was only associated with higher CSF levels of phosphorylated and Total Tau when the presence of the APOE e4 allele was considered (Starks et al., 2015). One of the mechanisms underlying this event involves the glycogen synthase kinase 3 β (GSK3β), a Tau kinase regulated by insulin *via* the AkT pathway (Welsh and Proud, 1993). Impaired brain insulin signaling, either as a result of insulin resistance due to the chronic exposure of neurons to high levels of insulin (Kim et al., 2011), or as a result of eventual decrease in brain insulin levels, triggers a reduction in AkT phosphorylation that leads to an increase in GSK3β activity and ultimately Tau phosphorylation (Zhang et al., 2018). Insulin can also affect phospho-Tau levels by decreasing the activity of Tau phosphatases (Gratuze et al., 2017). Protein phosphatase 2 (PP2A) is the primary Tau phosphatase implicated in AD, the activity of which is suppressed by insulin administration in humans and animals (Gong et al., 1995; Kins et al., 2001; Vogelsberg-Ragaglia et al., 2001). It remains to be investigated if defective insulin signaling, or tau pathology comes first in the disease pathogenesis. Our hypothesis is that it is individual specific. Regardless, in both cases, dysfunctional Tau protein and insulin signaling would aggravate each other and prompt or exacerbate cognitive decline in AD and other tauopathies.

In addition to insulin, insulin-like growth factors (IGFs) are important for the regulation of physiological functions such as glucose and energy metabolism, and they play crucial metabolic and neurotrophic roles in the brain (Gasparini and Xu, 2003; Sharma et al., 2016). The administration of IGFII in rats enhanced memory retention and prevented memory impairment (Chen et al., 2011). Serum levels of IGF1 reduces with aging and correlates with cognitive performance of individuals in the Mini Mental State Examination (MMSE) and in other neuropsychological assessments (Rollero et al., 1998; Kalmijn et al., 2000; Dik et al., 2003; Landi et al., 2007; Al-Delaimy et al., 2009; Angelini et al., 2009). Moreover, lower levels of IGF1 are both a risk factor and a feature of AD (Mustafa et al., 1999; Steen et al., 2005; Talbot et al., 2012; Westwood et al., 2014). Intriguingly, IGF proteins have been shown to regulate Tau phosphorylation *in vivo* (Schubert et al., 2003) and *in vitro* (Hong and Lee, 1997; Lesort et al., 1999; Lesort and Johnson, 2000), with the brain IGFI null mouse having increased hyperphosphorylated Tau when compared to WT animals (Cheng et al., 2005). Therefore, considering our hypothesis of Tau loss of function when this protein is hyperphosphorylated and aggregated in pathological conditions, it is likely that dysfunctional Tau is also a consequence of impaired IGF signaling in AD and other tauopathies. Moreover, because insulin and IGF signaling pathways share common intracellular components, it is possible that in disease, Tau loss of function affects IGF signaling. However, the association between the IGF system and Tau loss of function still needs to be investigated.

Apart from intracellular Tau, the existence of extracellular forms of Tau protein has been reported. The secretion of Tau *via* direct translocation across the plasma membrane was observed in neuroblastoma and in Chinese hamster ovary (CHO) cells overexpressing Tau constructs. In another study, Tau protein was released *via* exosomes by primary neuronal cultures and N2a cells overexpressing Tau constructs (Wang et al., 2017). While our current knowledge on the physiological functions of intracellular Tau is expanding, less is known about the role of extracellular Tau in physiology. A recent study has argued that the secretion and transfer of Tau between neurons is a physiological process rather than a disease-specific event (Evans et al., 2018). However, Tau secretion was shown to increase with Tau phosphorylation (Katsinelos et al., 2018), leading to the hypothesis that pathological Tau is secreted as a consequence of its reduced affinity to MTs when abnormally phosphorylated. Indeed, the exposure of neuroblastoma cells (SH-SY5Y) to conditioned medium from activated human microglia, caused an increase in the production and secretion of Tau (Lee et al., 2015), reinforcing the idea that neuroinflammation may impact Tau pathogenesis (Laurent et al., 2018). Therefore, with inflammation as a feature of the metabolic syndrome, a detrimental cycle might arise escalating both neurodegeneration and metabolic dysfunction in Tauopathies.

Neuronal-derived exosomes of total plasma from AD subjects presented increased levels of pIRS-1Ser312, a marker of insulin resistance, detected as early as 10 years before clinical onset of AD (Kapogiannis et al., 2015). In other studies, neuronal-derived exosomes in plasma of AD patients had higher levels of phosphorylated Tau (Abner et al., 2016), and the ratio of oligomeric to total Tau was higher in the CSF of AD patients (Sengupta et al., 2017). Despite the detection of markers of both Tau pathology and insulin resistance in exosomes derived from AD patients, it still remains to be elucidated how these species interact with each other regarding cause and consequence, their potential to act as trans-synaptic transmitter of pathology across neurons and their implication on brain function.

## Tau Protein and Insulin Signaling in Peripheral Tissues

A recent publication suggested that Tau in the brain can efflux into the blood and can be cleared in the periphery, and therefore, a chronic increase of peripheral Tau clearance can reduce pathological Tau accumulation, neurodegeneration, and neuroinflammation in the brain (Wang et al., 2018). Tau is cleared in kidney and liver under physiological conditions in both human and mice. Therefore, compromised kidney and liver function might impede Tau clearance from the brain and thereby contribute to neurodegeneration. Insulin resistance has been suggested to contribute to kidney dysfunction and may also lead to non-alcoholic fatty liver disease during type 2 diabetes (T2D; De Cosmo et al., 2013; Mohamed et al., 2016), providing an additional mechanism by which T2D may contribute to cognitive dysfunction.

While Tau has played a primary role in the pathogenesis of some neurodegenerative diseases and has been extensively studied in the context of the brain, Tau has only recently been proposed to play a role in peripheral metabolic regulation. Recent work by our group and others has shown that Tau is highly expressed in pancreatic islets, in insulin-secreting beta cells (Bharadwaj et al., 2017; Wijesekara et al., 2018). Interestingly, mice with a global KO of Tau show an increase in body weight and defects in glucose-stimulated insulin secretion and impaired glucose tolerance at a very young age (Wijesekara et al., 2018). A recent study also showed central insulin resistance in Tau KO animals, proposing that impaired insulin signaling in the hypothalamus, the main regulator of body weight, is a possibility. In accordance with this last hypothesis, Tau ablation inhibited the anorexigenic effects of insulin when delivered icv in mice (Marciniak et al., 2017). Mice with genetic deletion of insulin receptors in the brain [i.e., neuron-specific IR KO (NIRKO)] exhibit diet-sensitive obesity, increased food intake, and insulin resistance (Brüning et al., 2000). Moreover, brain insulin resistance typically results in increased hypothermia, augmented hepatic glucose output and impaired response to hypoglycemia (Kleinridders et al., 2014), all characteristics mirrored in the Tau KO animals, clearly suggesting the presence of reduced brain insulin signaling in these animals. While the effects on beta cell function could be a consequence of this, modulated by alterations in leptin levels, it was also evident that Tau plays a direct role in insulin production and secretion in these cells (Maj et al., 2016; Wijesekara et al., 2018). As a microtubule binding protein, Tau may be important for insulin granule movement to or sequestration at the plasma membrane mediating insulin secretion, and movement of proinsulin from the endoplasmic reticulum to the Golgi and subsequently to the insulin granule mediating insulin synthesis. In addition, one previous study suggested that Tau may also regulate insulin gene transcription (Maj et al., 2016).

Interestingly, elevated levels of Tau phosphorylation have been reported in the pancreas of patients with T2D (Miklossy et al., 2010), and recently, we showed significant Tau hyperphosphorylation in pancreatic islets from a transgenic mouse model of AD and T2D (Wijesekara et al., 2017). Whether this contributes to diabetes pathogenesis or is a consequence of diabetes itself remains to be understood. During diabetes, PI3 kinase pathway is typically down-regulated and therefore, GSK3 activity may be up-regulated, which could drive phosphorylation of Tau in diabetes (Hooper et al., 2008). Indeed, in pancreatic beta cell-derived rodent cell line, RIN-5F, inhibition of P13K was shown to increase Tau phosphorylation (Maj et al., 2016). Furthermore, it has been suggested that hypothermia, a consequence of insulin resistance could also drive Tau phosphorylation (El Khoury et al., 2016). Conversely, since hyperphosphorylated Tau may contribute to microtubule disassembly, changes in mitochondrial dynamics and inflammation (Beharry et al., 2014; Laurent et al., 2018), pathological Tau likely further contribute to beta cell dysfunction and insulin resistance. Regardless, the end result is an eventual reduction in insulin secretion, therefore, lower brain insulin levels leading to further aggravation of synaptic and cognitive impairments.

Although we were unable to observe appreciable expression of Tau in both mouse skeletal muscle and fat tissue (Wijesekara et al., 2018), its expression was shown in rat skeletal muscle in a previous study (Gu et al., 1996). An important feature of T2D is impaired glucose transport into skeletal muscle and adipose tissue, which is facilitated by GLUT4 glucose transporter translocation to the plasma membrane. While some have suggested that an intact microtubule system is important for the insulin-induced actin remodeling prior to the transporter translocation, some studies using agents that inhibit microtubule polymerization has suggested against this (Fletcher et al., 2000; Olson et al., 2001; Liu et al., 2013). Nonetheless, it has been shown that GLUT4 storage vesicles travel along microtubules *via* kinesins and actin filaments, from the perinuclear region, bringing them into close proximity with the plasma membrane SNARE proteins (Tunduguru and Thurmond, 2017). Newly formed GLUT4 vesicles also are transported from the plasma membrane to the cell interior by the microtubule-based motor protein dynein. However, it has been shown that heterologous overexpression of Tau protein, despite being localized to microtubules in 3T3-L1 adipocytes, delays the initial appearance of GLUT4 at the cell membrane following insulin stimulation (Emoto et al., 2001). This is in accordance with the view that excess Tau blocks axonal transport due to interference with motor proteins (Mandelkow et al., 2003). If Tau is indeed a critical regulator of GLUT4 movement in insulin-sensitive tissues, reduced glucose uptake into these tissues may have contributed to the early development of hyperglycemia in Tau KO mice and remains to be explored in future studies.

Current data suggests that there is clearly a role of Tau in peripheral tissue, particularly in regulating glucose homeostasis. However, the mechanisms linking central insulin resistance and peripheral Tau hyperphosphorylation and the metabolic consequences remain largely unknown. There are many factors that require consideration such as the impact of brain insulin resistance, particularly the hypothalamus on peripheral metabolic regulation, especially in the context of feeding behavior, body temperature regulation, energy expenditure; influence of pathological Tau on hormone regulation such as leptin and ghrelin and their impact on metabolism; direct changes to the microtubule system in individual tissues; and impact of gluco- and lipo-toxicity and peripheral insulin resistance and inflammation. Regardless, even with limited data, it is becoming quite clear that we cannot fully comprehend neurodegeneration without fully understanding the physiological roles of its key players such as Tau within the periphery.

## Conclusion

**Figure 1:**
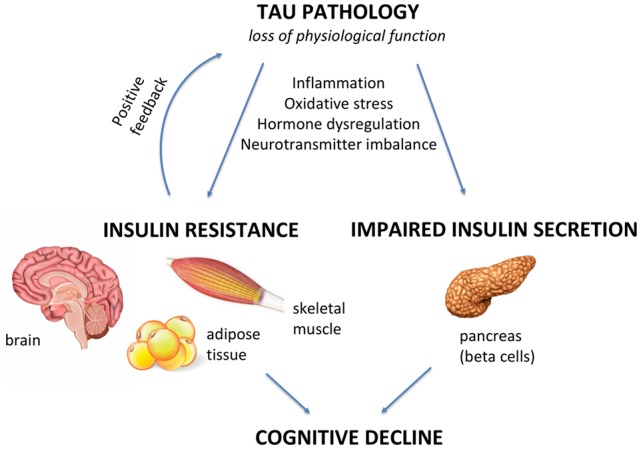
Tau pathology as a mediator of insulin resistance and cognitive and metabolic alterations. Under pathological conditions, Tau loss of function can negatively impact the brain and peripheral tissues, such as skeletal muscle, adipose tissue and pancreas. In this review article, we explore the hypothesis of Tau pathology being a key inductor of insulin resistance and beta cell dysfunction in Alzheimer’s disease (AD) and other tauopathies. When defective insulin signaling is present in the brain, it triggers neurodegeneration and cognitive decline. On the other hand, when it is present in peripheral tissues, it leads to a diabetes-like phenotype. Inflammation, oxidative stress, hormonal dysregulation and neurotransmitter imbalance are mechanisms that can lead to/aggravate Tau pathology-induced alterations in insulin signaling/secretion in the brain and in the periphery, which are further associated with cognitive defects.

Tau pathology has emerged as a trigger of insulin resistance and insulin deficiency in the brain and peripheral tissues, and it is suggested to be an early event in the pathogenesis of AD and other tauopathies, representing a promising therapeutic target capable of interfering with disease progression. Impaired insulin signaling can also trigger Tau pathology, sustaining a vicious cycle, with cognitive decline being the end result as illustrated in Figure 1. However, it remains to be elucidated whether impaired insulin signaling, or Tau pathology comes first in AD pathogenesis and other tauopathies.

The molecular mechanisms of how Tau impairs insulin signaling and insulin secretion also remains to be better investigated and might involve loss of Tau function or indirect mechanisms such as inflammation, oxidative stress or changes in hormone/neurotransmitter release. Regardless, it is becoming apparent that Tau may be an important link between neurodegeneration and diabetes and while Tau KO mice provide a good foundation for these studies, future experiments need to be geared towards understanding these phenomena at a whole-body level using transgenic Tau hyperphosphorylation models and tissue-specific Tau KO animals.

## Author Contributions

RG and FDF defined the topics to be covered and drafted the main part of the manuscript. NW participated in the conception and design of the figure, with contributions from RG. RG, NW, FDF and PF participated in the development and draft of the article, critical revision of the article and final approval of the version to be published.

## Conflict of Interest Statement

The authors declare that the research was conducted in the absence of any commercial or financial relationships that could be construed as a potential conflict of interest.

## Abbreviations

Aβ: Amyloid β
AD: Alzheimer’s disease
CSF: Cerebrospinal fluid
FTD: Frontotemporal dementia
GSK3β: Glycogen synthase kinase 3 β
NFTs: Neurofibrillary tangles
PHFs: Paired helical filaments
PTEN: Phosphatase and tensin homolog protein
PTMs: Post-translational modifications
AkT: Protein kinase B
SH3: Src homology 3
T2D: Type 2 diabetes.

## References

[B1] Abner E. L. Jicha G. A. Shaw L. M. Trojanowski J. Q. Goetzl E. J. (2016). Plasma neuronal exosomal levels of Alzheimer’s disease biomarkers in normal aging. Ann. Clin. Transl. Neurol. 3, 399–403. 10.1002/acn3.309 27231710 PMC4863753

[B2] Ahmed T. Van der Jeugd A. Blum D. Galas M. C. D’Hooge R. Buee L. (2014). Cognition and hippocampal synaptic plasticity in mice with a homozygous tau deletion. Neurobiol. Aging 35, 2474–2478. 10.1016/j.neurobiolaging.2014.05.005 24913895

[B3] Al-Delaimy W. K. von Muhlen D. Barrett-Connor E. (2009). Insulinlike growth factor-1, insulinlike growth factor binding protein-1 and cognitive function in older men and women. J. Am. Geriatr. Soc. 57, 1441–1446. 10.1111/j.1532-5415.2009.02343.x 19515112 PMC2728156

[B4] Angelini A. Bendini C. Neviani F. Bergamini L. Manni B. Trenti T.. (2009). Insulin-like growth factor-1 (IGF-1): relation with cognitive functioning and neuroimaging marker of brain damage in a sample of hypertensive elderly subjects. Arch. Gerontol. Geriatr. 49, 5–12. 10.1016/j.archger.2009.09.006 19836610

[B5] Beharry C. Cohen L. S. Di J. Ibrahim K. Briffa-Mirabella S. Alonso A. C. (2014). Tau-induced neurodegeneration: mechanisms and targets. Neurosci. Bull. 30, 346–358. 10.1007/s12264-013-1414-z 24733656 PMC5562659

[B6] Benedict C. Grillo C. A. (2018). Insulin resistance as a therapeutic target in the treatment of Alzheimer’s disease: a state-of-the-art review. Front. Neurosci. 12:215. 10.3389/fnins.2018.00215 29743868 PMC5932355

[B7] Bharadwaj P. Wijesekara N. Liyanapathirana M. Newsholme P. Ittner L. Fraser P.. (2017). The link between type 2 diabetes and neurodegeneration: roles for amyloid-β, amylin, and tau proteins. J. Alzheimers. Dis. 59, 421–432. 10.3233/JAD-161192 28269785

[B8] Biundo F. Del Prete D. Zhang H. Arancio O. D’Adamio L. (2018). A role for tau in learning, memory and synaptic plasticity. Sci. Rep. 8:3184. 10.1038/s41598-018-21596-3 29453339 PMC5816660

[B9] Bomfim T. R. Forny-Germano L. Sathler L. B. Brito-Moreira J. Houzel J. C. Decker H.. (2012). An anti-diabetes agent protects the mouse brain from defective insulin signaling caused by Alzheimer’s disease-associated Aβ oligomers. J. Clin. Invest. 122, 1339–1353. 10.1172/jci57256 22476196 PMC3314445

[B10] Brüning J. C. Gautam D. Burks D. J. Gillette J. Schubert M. Orban P. C.. (2000). Role of brain insulin receptor in control of body weight and reproduction. Science 289, 2122–2125. 10.1126/science.289.5487.2122 11000114

[B11] Chen D. Y. Stern S. A. Garcia-Osta A. Saunier-Rebori B. Pollonini G. Bambah-Mukku D.. (2011). A critical role for IGF-II in memory consolidation and enhancement. Nature 469, 491–497. 10.1038/nature09667 21270887 PMC3908455

[B12] Cheng C. M. Tseng V. Wang J. Wang D. Matyakhina L. Bondy C. A. (2005). Tau is hyperphosphorylated in the insulin-like growth factor-I null brain. Endocrinology 146, 5086–5091. 10.1210/en.2005-0063 16123158

[B13] Craft S. Baker L. D. Montine T. J. Minoshima S. Watson G. S. Claxton A.. (2012). Intranasal insulin therapy for Alzheimer disease and amnestic mild cognitive impairment: a pilot clinical trial. Arch. Neurol. 69, 29–38. 10.1001/archneurol.2011.233 21911655 PMC3260944

[B14] Craft S. Claxton A. Baker L. D. Hanson A. J. Cholerton B. Trittschuh E. H.. (2017). Effects of regular and long-acting insulin on cognition and Alzheimer’s disease biomarkers: a pilot clinical trial. J. Alzheimers Dis. 57, 1325–1334. 10.3233/jad-161256 28372335 PMC5409050

[B15] De Cosmo S. Menzaghi C. Prudente S. Trischitta V. (2013). Role of insulin resistance in kidney dysfunction: insights into the mechanism and epidemiological evidence. Nephrol. Dial. Transplant 28, 29–36. 10.1093/ndt/gfs290 23048172

[B16] De Felice F. G. (2013). Alzheimer’s disease and insulin resistance: translating basic science into clinical applications. J. Clin. Invest. 123, 531–539. 10.1172/jci64595 23485579 PMC3561831

[B17] Deng Y. Li B. Liu Y. Iqbal K. Grundke-Iqbal I. Gong C.-X. (2009). Dysregulation of insulin signaling, glucose transporters, *O*-GlcNAcylation, and phosphorylation of tau and neurofilaments in the brain: implication for Alzheimer’s disease. Am. J. Pathol. 175, 2089–2098. 10.2353/ajpath.2009.090157 19815707 PMC2774072

[B18] Dik M. G. Pluijm S. M. Jonker C. Deeg D. J. Lomecky M. Z. Lips P. (2003). Insulin-like growth factor I (IGF-I) and cognitive decline in older persons. Neurobiol. Aging 24, 573–581. 10.1016/S0197-4580(02)00136-7 12714114

[B19] Duarte A. I. Moreira P. I. Oliveira C. R. (2012). Insulin in central nervous system: more than just a peripheral hormone. J. Aging Res. 2012:384017. 10.1155/2012/384017 22500228 PMC3303591

[B20] El Khoury N. B. Gratuze M. Papon M. A. Bretteville A. Planel E. (2014). Insulin dysfunction and tau pathology. Front. Cell. Neurosci 8:22. 10.3389/fncel.2014.00022 24574966 PMC3920186

[B21] El Khoury N. B. Gratuze M. Petry F. Papon M.-A. Julien C. Marcouiller F.. (2016). Hypothermia mediates age-dependent increase of tau phosphorylation in db/db mice. Neurobiol. Dis. 88, 55–65. 10.1016/j.nbd.2016.01.005 26777665

[B22] Emoto M. Langille S. E. Czech M. P. (2001). A role for kinesin in insulin-stimulated GLUT4 glucose transporter translocation in 3T3-L1 adipocytes. J. Biol. Chem. 276, 10677–10682. 10.1074/jbc.M010785200 11145966

[B23] Evans L. D. Wassmer T. Fraser G. Smith J. Perkinton M. Billinton A.. (2018). Extracellular monomeric and aggregated tau efficiently enter human neurons through overlapping but distinct pathways. Cell Rep. 22, 3612–3624. 10.1016/j.celrep.2018.03.021 29590627 PMC5896171

[B24] Fernandez A. M. Torres-Alemán I. (2012). The many faces of insulin-like peptide signalling in the brain. Nat. Rev. Neurosci. 13, 225–239. 10.1038/nrn3209 22430016

[B25] Fletcher L. M. Welsh G. I. Oatey P. B. Tavaré J. M. (2000). Role for the microtubule cytoskeleton in GLUT4 vesicle trafficking and in the regulation of insulin-stimulated glucose uptake. Biochem. J. 352, 267–276. 10.1042/bj3520267 11085918 PMC1221456

[B27] Gasparini L. Xu H. (2003). Potential roles of insulin and IGF-1 in Alzheimer’s disease. Trends Neurosci. 26, 404–406. 10.1016/s0166-2236(03)00163-2 12900169

[B29] Goedert M. Spillantini M. G. (2011). Pathogenesis of the tauopathies. J. Mol. Neurosci. 45, 425–431. 10.1007/s12031-011-9593-4 21785996

[B30] Gong C. X. Shaikh S. Wang J.-Z. Zaidi T. Grundke-Iqbal I. Iqbal K. (1995). Phosphatase activity toward abnormally phosphorylated tau: decrease in Alzheimer disease brain. J. Neurochem. 65, 732–738. 10.1046/j.1471-4159.1995.65020732.x 7616230

[B31] Gratuze M. Julien J. Petry F. R. Morin F. Planel E. (2017). Insulin deprivation induces PP2A inhibition and tau hyperphosphorylation in hTau mice, a model of Alzheimer’s disease-like tau pathology. Sci. Rep. 7:46359. 10.1038/srep46359 28402338 PMC5389355

[B32] Grundke-Iqbal I. Iqbal K. Quinlan M. Tung Y. C. Zaidi M. S. Wisniewski H. M. (1986). Microtubule-associated protein tau. A component of Alzheimer paired helical filaments. J. Biol. Chem. 261, 6084–6089. 3084478

[B33] Gu Y. Oyama F. Ihara Y. (1996). τ is widely expressed in rat tissues. J. Neurochem. 67, 1235–1244. 10.1046/j.1471-4159.1996.67031235.x 8752131

[B34] Guo T. Noble W. Hanger D. P. (2017). Roles of tau protein in health and disease. Acta Neuropathol. 133, 665–704. 10.1007/s00401-017-1707-9 28386764 PMC5390006

[B35] Hong M. Lee V. M.-Y. (1997). Insulin and insulin-like growth factor-1 regulate tau phosphorylation in cultured human neurons. J. Biol. Chem. 272, 19547–19553. 10.1074/jbc.272.31.19547 9235959

[B36] Hooper C. Killick R. Lovestone S. (2008). The GSK3 hypothesis of Alzheimer’s disease. J. Neurochem. 104, 1433–1439. 10.1111/j.1471-4159.2007.05194.x 18088381 PMC3073119

[B37] Kalmijn S. Janssen J. A. M. J. L. Pols H. A. P. Lamberts S. W. Breteler M. M. (2000). A prospective study on circulating insulin-like growth factor I (IGF-I), IGF-binding proteins, and cognitive function in the elderly. J. Clin. Endocrinol. Metab. 85, 4551–4555. 10.1210/jc.85.12.4551 11134107

[B38] Kapogiannis D. Boxer A. Schwartz J. B. Abner E. L. Biragyn A. Masharani U.. (2015). Dysfunctionally phosphorylated type 1 insulin receptor substrate in neural-derived blood exosomes of preclinical Alzheimer’s disease. FASEB J. 29, 589–596. 10.1096/fj.14-262048 25342129 PMC4314222

[B39] Katsinelos T. Zeitler M. Dimou E. Karakatsani A. Müller H. M. Nachman E.. (2018). Unconventional secretion mediates the trans-cellular spreading of tau. Cell Rep. 23, 2039–2055. 10.1016/j.celrep.2018.04.056 29768203

[B40] Kim B. Sullivan K. A. Backus C. Feldman E. L. (2011). Cortical neurons develop insulin resistance and blunted Akt signaling: a potential mechanism contributing to enhanced ischemic injury in diabetes. Antioxid. Redox Signal. 14, 1829–1839. 10.1089/ars.2010.3816 21194385 PMC3078499

[B41] Kins S. Crameri A. Evans D. R. H. Hemmings B. A. Nitsch R. M. Götz J. (2001). Reduced protein phosphatase 2A activity induces hyperphosphorylation and altered compartmentalization of tau in transgenic mice. J. Biol. Chem. 276, 38193–38200. 10.1074/jbc.M102621200 11473109

[B42] Kleinridders A. Ferris H. A. Cai W. Kahn C. R. (2014). Insulin action in brain regulates systemic metabolism and brain function. Diabetes 63, 2232–2243. 10.2337/db14-0568 24931034 PMC4066341

[B43] Kuga G. K. Muñoz V. R. Gaspar R. C. Nakandakari S. C. B. R. da Silva A. S. R. Botezelli J. D.. (2018). Impaired insulin signaling and spatial learning in middle-aged rats: the role of PTP1B. Exp. Gerontol. 104, 66–71. 10.1016/j.exger.2018.02.005 29421605

[B44] Landi F. Capoluongo E. Russo A. Onder G. Cesari M. Lulli P.. (2007). Free insulin-like growth factor-I and cognitive function in older persons living in community. Growth Horm. IGF Res. 17, 58–66. 10.1016/j.ghir.2006.11.001 17208483

[B46] Laurent C. Buée L. Blum D. (2018). Tau and neuroinflammation: what impact for Alzheimer’s disease and tauopathies? Biomed. J. 41, 21–33. 10.1016/j.bj.2018.01.003 29673549 PMC6138617

[B47] Laws S. M. Gaskin S. Woodfield A. Srikanth V. Bruce D. Fraser P. E.. (2017). Insulin resistance is associated with reductions in specific cognitive domains and increases in CSF tau in cognitively normal adults. Sci. Rep. 7:9766. 10.1038/s41598-017-09577-4 28852028 PMC5575049

[B48] Lee M. McGeer E. McGeer P. L. (2015). Activated human microglia stimulate neuroblastoma cells to upregulate production of beta amyloid protein and tau: implications for Alzheimer’s disease pathogenesis. Neurobiol. Aging 36, 42–52. 10.1016/j.neurobiolaging.2014.07.024 25169677

[B49] Lesort M. Johnson G. V. W. (2000). Insulin-like growth factor-1 and insulin mediate transient site-selective increases in tau phosphorylation in primary cortical neurons. Neuroscience 99, 305–316. 10.1016/s0306-4522(00)00200-1 10938436

[B50] Lesort M. Jope R. S. Johnson G. V. (1999). Insulin transiently increases tau phosphorylation: involvement of glycogen synthase kinase-3beta and Fyn tyrosine kinase. J. Neurochem. 72, 576–584. 10.1046/j.1471-4159.1999.0720576.x 9930729

[B51] Liu L.-Z. Cheung S. C. Lan L.-L. Ho S. K. Chan J. C. Tong P. C. (2013). Microtubule network is required for insulin-induced signal transduction and actin remodeling. Mol. Cell. Endocrinol. 365, 64–74. 10.1016/j.mce.2012.09.005 22996137

[B52] Lourenco M. V. Ferreira S. T. De Felice F. G. (2015). Neuronal stress signaling and eIF2α phosphorylation as molecular links between Alzheimer’s disease and diabetes. Prog. Neurobiol. 129, 37–57. 10.1016/j.pneurobio.2015.03.003 25857551

[B54] Maj M. Hoermann G. Rasul S. Base W. Wagner L. Attems J. (2016). The microtubule-associated protein tau and its relevance for pancreatic beta cells. J. Diabetes Res. 2016:1964634. 10.1155/2016/1964634 26824039 PMC4707345

[B55] Mandelkow E. M. Stamer K. Vogel R. Thies E. Mandelkow E. (2003). Clogging of axons by tau, inhibition of axonal traffic and starvation of synapses. Neurobiol. Aging 24, 1079–1085. 10.1016/j.neurobiolaging.2003.04.007 14643379

[B56] Marciniak E. Leboucher A. Caron E. Ahmed T. Tailleux A. Dumont J.. (2017). Tau deletion promotes brain insulin resistance. J. Exp. Med. 214, 2257–2269. 10.1084/jem.20161731 28652303 PMC5551570

[B57] Meier S. Bell M. Lyons D. N. Ingram A. Chen J. Gensel J. C.. (2015). Identification of novel tau interactions with endoplasmic reticulum proteins in Alzheimer’s disease brain. J. Alzheimers Dis. 48, 687–702. 10.3233/jad-150298 26402096 PMC4881838

[B58] Miklossy J. Qing H. Radenovic A. Kis A. Vileno B. Làszló F.. (2010). Beta amyloid and hyperphosphorylated tau deposits in the pancreas in type 2 diabetes. Neurobiol. Aging 31, 1503–1515. 10.1016/j.neurobiolaging.2008.08.019 18950899 PMC4140193

[B59] Mohamed J. Nazratun Nafizah A. H. Zariyantey A. H. Budin S. B. (2016). Mechanisms of diabetes-induced liver damage: the role of oxidative stress and inflammation. Sultan Qaboos Univ. Med. J. 16, e132–e141. 10.18295/squmj.2016.16.02.002 27226903 PMC4868511

[B60] Moloney A. M. Griffin R. J. Timmons S. O’Connor R. Ravid R. O’Neill C. (2010). Defects in IGF-1 receptor, insulin receptor and IRS-1/2 in Alzheimer’s disease indicate possible resistance to IGF-1 and insulin signalling. Neurobiol. Aging 31, 224–243. 10.1016/j.neurobiolaging.2008.04.002 18479783

[B61] Mustafa A. Lannfelt L. Lilius L. Islam A. Winblad B. Adem A. (1999). Decreased plasma insulin-like growth factor-I level in familial Alzheimer’s disease patients carrying the Swedish APP 670/671 mutation. Dement Geriatr. Cogn. Disord. 10, 446–451. 10.1159/000017188 10559558

[B63] Olson A. L. Trumbly A. R. Gibson G. V. (2001). Insulin-mediated GLUT4 translocation is dependent on the microtubule network. J. Biol. Chem. 276, 10706–10714. 10.1074/jbc.m007610200 11278355

[B64] Papegaey A. Eddarkaoui S. Deramecourt V. Fernandez-Gomez F. J. Pantano P. Obriot H.. (2016). Reduced Tau protein expression is associated with frontotemporal degeneration with progranulin mutation. Acta Neuropathol. Commun. 4:74. 10.1186/s40478-016-0345-0 27435172 PMC4952067

[B65] Reynolds C. H. Garwood C. J. Wray S. Price C. Kellie S. Perera T.. (2008). Phosphorylation regulates tau interactions with Src homology 3 domains of phosphatidylinositol 3-kinase, phospholipase Cgamma1, Grb2, and Src family kinases. J. Biol. Chem. 283, 18177–18186. 10.1074/jbc.m709715200 18467332

[B66] Rodriguez-Rodriguez P. Sandebring-Matton A. Merino-Serrais P. Parrado-Fernandez C. Rabano A. Winblad B.. (2017). Tau hyperphosphorylation induces oligomeric insulin accumulation and insulin resistance in neurons. Brain 140, 3269–3285. 10.1093/brain/awx256 29053786

[B67] Rollero A. Murialdo G. Fonzi S. Garrone S. Gianelli M. V. Gazzerro E.. (1998). Relationship between cognitive function, growth hormone and insulin-like growth factor I plasma levels in aged subjects. Neuropsychobiology 38, 73–79. 10.1159/000026520 9732206

[B68] Schubert M. Brazil D. P. Burks D. J. Kushner J. A. Ye J. Flint C. L.. (2003). Insulin receptor substrate-2 deficiency impairs brain growth and promotes tau phosphorylation. J. Neurosci. 23, 7084–7092. 10.1523/jneurosci.23-18-07084.2003 12904469 PMC6740672

[B69] Sengupta U. Portelius E. Hansson O. Farmer K. Castillo-Carranza D. Woltjer R.. (2017). Tau oligomers in cerebrospinal fluid in Alzheimer’s disease. Ann. Clin. Transl. Neurol. 4, 226–235. 10.1002/acn3.382 28382304 PMC5376754

[B71] Sharma A. N. da Costa e Silva B. F. Soares J. C. Carvalho A. F. Quevedo J. (2016). Role of trophic factors GDNF, IGF-1 and VEGF in major depressive disorder: a comprehensive review of human studies. J. Affect Disord. 197, 9–20. 10.1016/j.jad.2016.02.067 26956384 PMC4837031

[B72] Starks E. J. Patrick O’Grady J. Hoscheidt S. M. Racine A. M. Carlsson C. M. Zetterberg H.. (2015). Insulin resistance is associated with higher cerebrospinal fluid tau levels in asymptomatic APOEε4 carriers. J. Alzheimers Dis. 46, 525–533. 10.3233/jad-150072 25812851 PMC4583335

[B73] Steen E. Terry B. M. Rivera E. J. Cannon J. L. Neely T. R. Tavares R.. (2005). Impaired insulin and insulin-like growth factor expression and signaling mechanisms in Alzheimer’s disease–is this type 3 diabetes? J. Alzheimers Dis. 7, 63–80. 10.3233/jad-2005-7107 15750215

[B74] Stefanoska K. Volkerling A. Bertz J. Poljak A. Ke Y. D. Ittner L. M.. (2018). An N-terminal motif unique to primate tau enables differential protein-protein interactions. J. Biol. Chem. 293, 3710–3719. 10.1074/jbc.ra118.001784 29382714 PMC5846163

[B75] Suzuki M. Kimura T. (2017). Microtubule-associated tau contributes to intra-dendritic trafficking of AMPA receptors in multiple ways. Neurosci. Lett. 653, 276–282. 10.1016/j.neulet.2017.05.056 28554859

[B76] Talbot K. Wang H. Y. Kazi H. Han L. Y. Bakshi K. P. Stucky A.. (2012). Demonstrated brain insulin resistance in Alzheimer’s disease patients is associated with IGF-1 resistance, IRS-1 dysregulation, and cognitive decline. J. Clin. Invest. 122, 1316–13138. 10.1172/jci59903 22476197 PMC3314463

[B77] Tunduguru R. Thurmond D. C. (2017). Promoting glucose transporter-4 vesicle trafficking along cytoskeletal tracks: PAK-Ing them out. Front. Endocrinol. 8:329. 10.3389/fendo.2017.00329 29209279 PMC5701999

[B78] Vogelsberg-Ragaglia V. Schuck T. Trojanowski J. Q. Lee V. M. (2001). PP2A mRNA expression is quantitatively decreased in Alzheimer’s disease hippocampus. Exp. Neurol. 168, 402–412. 10.1006/exnr.2001.7630 11259128

[B79] Wang J. Jin W. S. Bu X. L. Zeng F. Huang Z. L. Li W. W.. (2018). Physiological clearance of tau in the periphery and its therapeutic potential for tauopathies. Acta Neuropathol. 136, 525–536. 10.1007/s00401-018-1891-2 30074071

[B80] Wang Y. Balaji V. Kaniyappan S. Krüger L. Irsen S. Tepper K.. (2017). The release and trans-synaptic transmission of Tau via exosomes. Mol. Neurodegener. 12:5. 10.1186/s13024-016-0143-y 28086931 PMC5237256

[B82] Weingarten M. D. Lockwood A. H. Hwo S. Y. Kirschner M. W. (1975). A protein factor essential for microtubule assembly. Proc. Natl. Acad. Sci. U S A 72, 1858–1862. 1057175 10.1073/pnas.72.5.1858 PMC432646

[B83] Welsh G. I. Proud C. G. (1993). Glycogen synthase kinase-3 is rapidly inactivated in response to insulin and phosphorylates eukaryotic initiation factor eIF-2B. Biochem. J. 294, 625–629. 10.1042/bj2940625 8397507 PMC1134506

[B84] Westwood A. J. Beiser A. Decarli C. Harris T. B. Chen T. C. He X. M.. (2014). Insulin-like growth factor-1 and risk of Alzheimer dementia and brain atrophy. Neurology 82, 1613–1619. 10.1212/wnl.0000000000000382 24706014 PMC4013812

[B85] Wijesekara N. Ahrens R. Sabale M. Wu L. Ha K. Verdile G. M.. (2017). Amyloid-β and islet amyloid pathologies link Alzheimer disease and type 2 diabetes in a transgenic model. FASEB J. 31, 5409–5418. 10.1096/fj.201700431R 28808140

[B86] Wijesekara N. Gonçalves R. A. Ahrens R. De Felice F. G. Fraser P. E. (2018). Tau ablation in mice leads to pancreatic β cell dysfunction and glucose intolerance. FASEB J. 32, 3166–3173. 10.1096/fj.201701352 29401605

[B87] Yarchoan M. Toledo J. B. Lee E. B. Arvanitakis Z. Kazi H. Han L. Y.. (2014). Abnormal serine phosphorylation of insulin receptor substrate 1 is associated with tau pathology in Alzheimer’s disease and tauopathies. Acta Neuropathol. 128, 679–689. 10.1007/s00401-014-1328-5 25107476 PMC4304658

[B88] Zhang Y. Huang N. Q. Yan F. Jin H. Zhou S. Y. Shi J. S.. (2018). Diabetes mellitus and Alzheimer’s disease: GSK-3β as a potential link. Behav. Brain Res. 339, 57–65. 10.1016/j.bbr.2017.11.015 29158110

[B89] Zhou L. McInnes J. Wierda K. Holt M. Herrmann A. G. Jackson R. J.. (2017). Tau association with synaptic vesicles causes presynaptic dysfunction. Nat. Commun. 8:15295. 10.1038/ncomms15295 28492240 PMC5437271

[B90] Zhukareva V. Sundarraj S. Mann D. Sjogren M. Blenow K. Clark C. M.. (2003). Selective reduction of soluble tau proteins in sporadic and familial frontotemporal dementias: an international follow-up study. Acta Neuropathol. 105, 469–476. 10.1007/s00401-002-0668-8 12677447

